# GourdBase: a genome-centered multi-omics database for the bottle gourd (*Lagenaria siceraria*), an economically important cucurbit crop

**DOI:** 10.1038/s41598-018-22007-3

**Published:** 2018-02-26

**Authors:** Ying Wang, Pei Xu, Xiaohua Wu, Xinyi Wu, Baogen Wang, Yunping Huang, Yaowen Hu, Jiandong Lin, Zhongfu Lu, Guojing Li

**Affiliations:** 1 Institute of Vegetables, Zhejiang Academy of Agricultural Sciences, Hangzhou, 310021 PR China; 2 Institute of Vegetables, Ningbo Academy of Agricultural Sciences, Ningbo, 315040 China

## Abstract

GourdBase is an integrative data platform for the bottle gourd to examine its multifarious intuitive morphology and annotated genome. GourdBase consists of six main modules that store and interlink multi-omic data: the genome (with transcriptomic data integrated) module, the phenome module, the markers/QTLs module, the maps (genetic, physical and comparative) module, the cultivars module, and the publications module. These modules provide access to various type of data including the annotated reference genome sequence, gene models, transcriptomic data from various tissues, physical and comparative genome maps, molecular markers in different types, phenotypic data for featuring traits including fruit shape and umami taste, and quantitative trait loci (QTLs) that underlie these traits. GourdBase is intuitive, user-friendly and interlinked and is designed to allow researchers, breeders and trained farmers to browse, search and fetch information on interests and assist in genomics-driven studies and breeding. The knowledge base and web interface can be accessed at http://www.gourdbase.cn/.

## Introduction

The bottle gourd [*Lagenaria siceraria* (Mol.) Standl.] (2n = 2x = 22), native to Africa and believed to be independently domesticated in Africa and Asia^1^, is an important horticultural and medicinal crop that belongs to the *Cucurbitaceae* family^1–3^. The fruit is the primary economic organ of the bottle gourd, which can be used as a vegetable, a container, a decoration item or an instrument^4,5^.

Due to the rapid progress of genomic technologies^6,7^, obtaining a high-quality genome assembly is no longer a bottleneck for most of the plant species. We previously reported on the first dense genetic map and framework genome of the bottle gourd^8^ and have recently assembled the entire genome for the Chinese bottle gourd landrace “Hangzhou gourd” for food use. We have also generated other types of omics data including spatial transcriptomic profiles and gene co-expression networks. However, precise and high-throughput phenotyping still remains a challenge. For example, fruit shape is a composite and complex trait controlled by multiple QTLs^9–13^. Although tremendous genetic diversity in fruit shapes exists in the bottle gourd germplasm^8^, only a few genes responsible for fruit shape have been identified thus far. *SUN*, *OVATE*, *WOX and YABBY* are the genes that have been cloned and characterized for determining fruit shape in the fleshy crop tomato model^14–17^. An *OVATE-*like gene, *CaOVATE*, was shown to control fruit shape in pepper^18^. Although many fruit shape QTLs were mapped in major cucurbit crops, including in melons, cucumbers and watermelons^9–13^, little is known about the genes underlying these QTLs.

Fruit taste quality is another important agricultural trait of the bottle gourd that is difficult to dissect. Cultivars with a peculiar umami taste in fruit are more favored in the market. In many vegetables, free amino acids are recognized to be the umami ingredients. For example, tomato fruit ripening was reported to be accompanied by an increase of the concentration of amino acids. As a result, fully ripened tomato fruits usually contain higher levels of free glutamic acid^19,20^. Free glutamate is the main ingredient responsible for the umami taste in edible mushrooms^21^. However, which components contribute to the umami taste of bottle gourd fruits is unclear.

In addition, with the rapid expansion of bioinformatics data, how these data should be stored and interlinked and displayed in an interface requires special efforts. From a breeder’s perspective, easy-to-use tools such as a marker browser, genetic maps with information on trait QTLs and designing tools for marker-assisted selection are needed. At present, there are two web servers for the cucurbits that are publically available, including the Cucurbit Genomics Database (CGD, http://cucurbitgenomics.org/), and the Cucumber Genome DataBase (http://cucumber.genomics.org.cn/page/cucumber/index.jsp). However, only CGD holds a section for the bottle gourd, in which the genome sequence of the *Lagenaria siceraria* cv. USVL1VR-Ls is retrievable. Other information that is essential for molecular breeding, such as phenotypic information and gene regulatory markers is missing. Therefore, there is an urgent need to construct a dedicated and more comprehensive bottle gourd multi-omics database to allows researchers, breeders and trained farmers to search and access various type of data.

In this study, a dedicated database named GourdBase was developed to assist in the study of biological traits and molecular breeding in the bottle gourd. The main purpose of GourdBase is to provide a reference genome resource (annotation genes, transcriptomics and proteins), a phenome (fruit shape and umami taste phenotypes), molecular markers (InDels, SSRs, SNPs), and maps (genetic, physical and comparative) to enable marker-assisted breeding. GourdBase also features hundreds of fruit shape images, secondary geometric fruit shape parameters and QTLs, which make the database valuable for studying fruit shapes.

## Results

### The database contents of GourdBase

**Figure 1:**
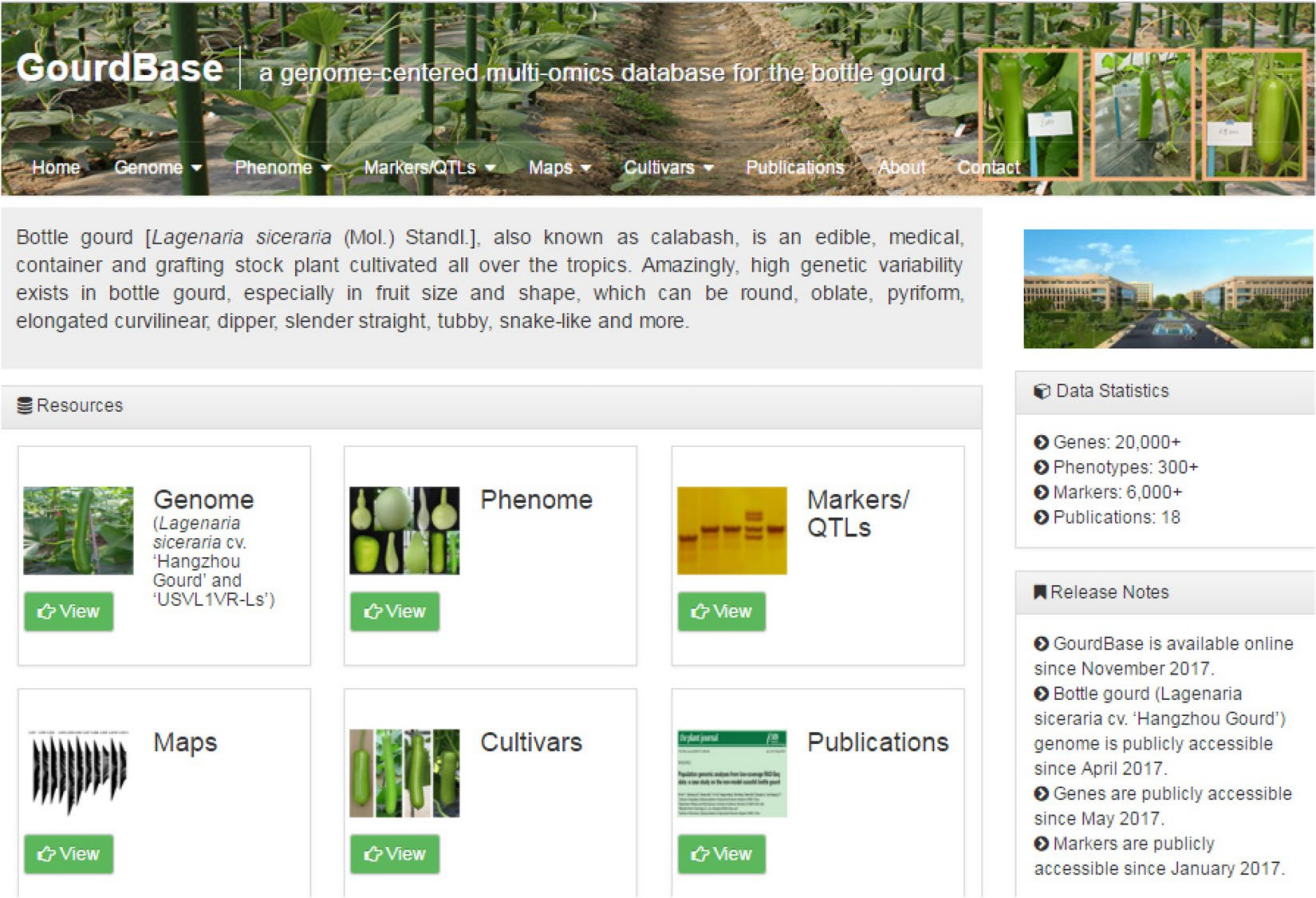
The GourdBase homepage. Six main modules are displayed in this interface and include the following: the genome (with transcriptomic data integrated), phenome, markers/QTLs, maps (genetic, physical and comparative), released cultivars, and publications modules.

**Figure 2:**
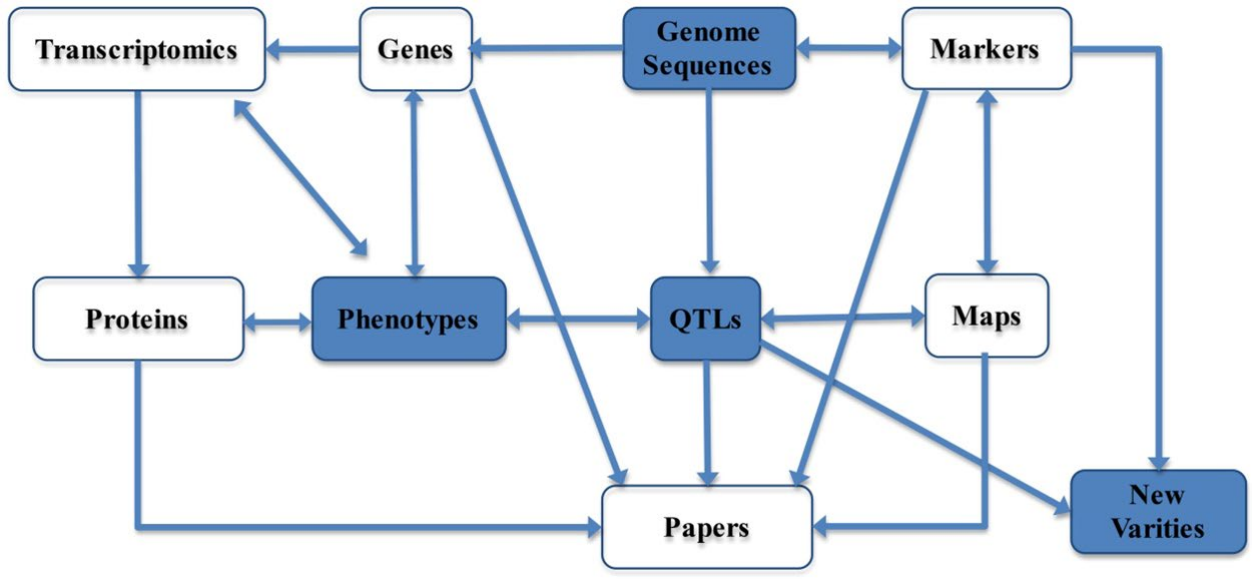
A flow diagram showing the interlink of all of the modules and datasets integrated into the GourdBase.

GourdBase consists of six main modules: the genome (with transcriptomic data integrated) module, the phenome module, the markers/QTLs module, the maps (genetic, physical and comparative) module, the released cultivars module, and the publications module (Fig. 1). Currently, GourdBase contains the following information: 27,500 genes (25,502 protein-coding genes) with tissue expression patterns; over 6000 various types of molecular markers (SNPs, InDels, SSR, AFLP, etc.); fruit shape phenotypes for 137 diverse accessions, umami taste phenotypes that are expressed as free amino acid contents for 135 accessions^22^; 56 QTLs for various traits including 17 umami taste-related QTLs, 37 fruit shape-related QTLs and 2 bitterness taste-related QTLs; 4 genetic, physical and comparative maps; 4 released cultivars with detailed information. GourdBase also harbors a publications module in which currently 18 publications supporting the aforementioned information can be freely read and downloaded with the authors’ permission. All of the modules and datasets in the GourdBase combine and interrelate with one another (Fig. 2).

### The application of the web interface

#### Genome Search

**Figure 3:**
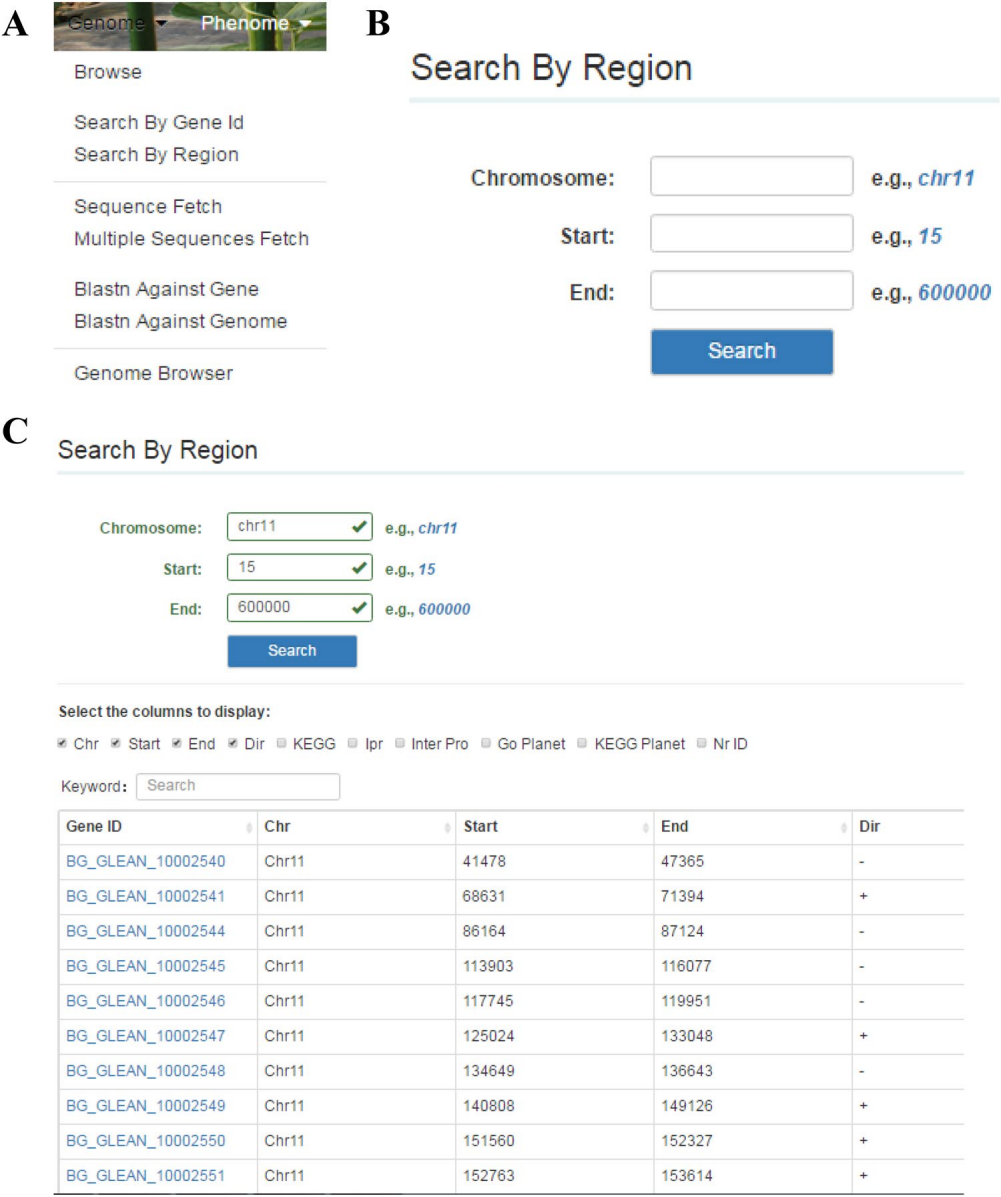
A detailed view of the ‘Genome’ function. (**A**) ‘Genome’ consists of ‘Browse’, ‘Search By Gene Id’, ‘Search By Region’, ‘Sequence Fetch’, ‘Multiple Sequences Fetch’, ‘Blastn Against Gene’, ‘Blastn Against Genome’ and ‘Genome Browser’. (**B**) Demonstration of the ‘Search By Region’ dialog box. (**C**) An example of the search result.

The GourdBase genome database provides publicly available information for the recently assembled genome of the Chinese bottle gourd landrace ‘Hangzhou Gourd’ in the authors’ lab and of the Indian accession ‘cv. USVL1VR-Ls’ that was initially released in the CGD. We predicted 25,502 protein-coding genes in the ‘Hangzhou Gourd’ genome, exceeding what was predicted in the watermelon (23,440, east Asia watermelon cultivar 97103) and cucumber (23,248, *Cucumis sativus var*. *sativus*) genomes^23,24^. When one drags the mouse over the ‘Genome’, the column header label appears, which reads ‘Browse’, ‘Search By Gene Id’, ‘Search By Region’, ‘Sequence Fetch’, ‘Multiple Sequences Fetch’, ‘Blastn Against Gene’, ‘Blastn Against Genome’ and ‘Genome Browser’ (Fig. 3A). The user can click on any one of these labels to the sublinks and search for the information needed. For example, if one clicks on ‘Search By Region’, then an extra layer appears that reads ‘Chromosome’, ‘Start’, and ‘End’ (Fig. 3B). The user can fill the query information in the box as demonstrated, and when he or she clicks on ‘Search’, large amounts of data appear quickly as requested (Fig. 3C). In the GourdBase genome database, the Basic Local Alignment Search Tool (BLAST) is also available to align sequences to determine similarities. ‘Blast Sequence Alignment Query’, ‘Blast Against Gene’ and ‘Blast Against Genome’ allow one to search the diverse genome resources.

#### Genome Browser

**Figure 4:**
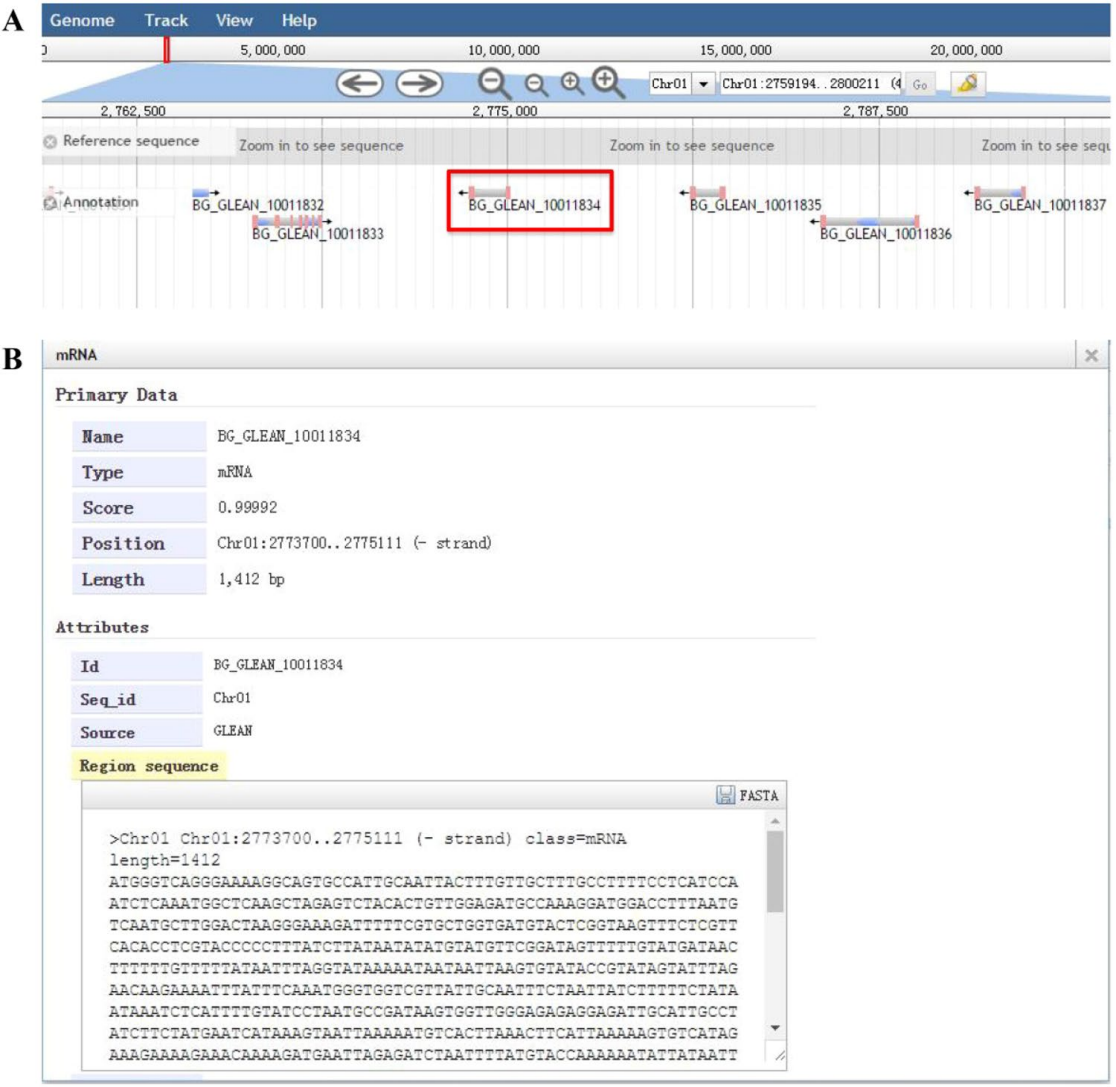
A regional view of the genome using the GourdBase genome browser. (**A**) A graphic view of the region 2759194 bp to 2800211 bp on Chr.1. (**B**) The interface after clicking on ‘BG_GLEAN_10011834’.

The genome browser of GourdBase includes tracks describing genes, mRNAs, peptides, and other features of interest and provides a graphical display of annotations on the bottle gourd genome (Fig. 4). Users can browse gene models on chromosomes and unanchored contigs and scaffolds. For example, if one sets up the genomic region of 2759194 bp to 2800211 bp on Chr.1 for browsing, all of the genes in this region will appear in order (Fig. 4A). When clicking on ‘BG_GLEAN_10011834’, an extra layer will appear with the detailed information such as mRNAs, CDS, peptides, and other features (Fig. 4B).

#### Transcriptomic data

The transcriptomic database of GourdBase stores and displays different RNA-seq datasets from different tissues including roots, leaves, ovaries, and the tender fruits of ‘Hangzhou Gourd’. Currently, the transcriptomics data has been integrated into the GourdBase genome database, which provides a simple platform for visualizing the expression data of one particular gene and as part of the detailed information of this gene in the genome module. The RNA-Seq information is shown at the bottom of each gene information page in the form of box-illustrations.

#### Phenotypic data

**Figure 5:**
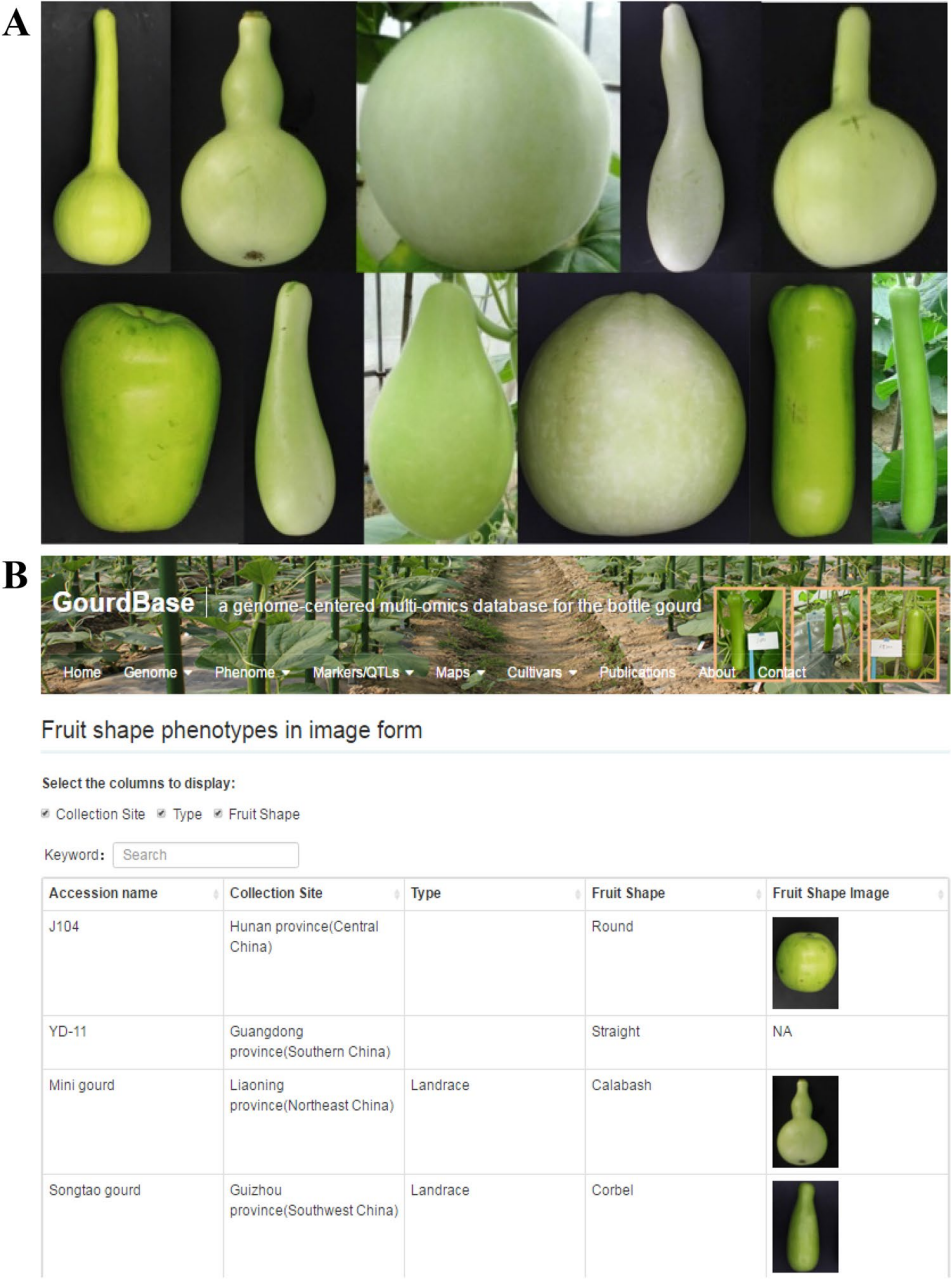
Shape diversity of bottle gourd fruits. (**A**) Highly variable fruit shapes in the bottle gourd germplasm pool. (**B**) A snapshot of the GourdBase phenome database.

The GourdBase phenome database currently contains the following data: fruit shape phenotypes in image form, fruit shape phenotypes in digital form, and umami taste phenotypes in numeric form. Highly variable fruit shapes are found in the bottle gourd germplasm, and the majority of the fruit shape types are shown in the phenome database of GourdBase (Fig. 5A). The image data covers the fruit shapes of round, pyriform, hulu (double-gourd), slender straight, corbel, long handled round, and tubby, to name a few. The image also provides information on the name, site of origin, and types for 137 bottle gourd accessions (Fig. 5B). In addition, to acquire more precise data on fruit shapes, we digitalized the fruit images to extract the shape outline and used the Elliptic Fourier analysis to fit the outline of each shape. The simulated shape outlines were overlaid and displayed in the fruit shape phenotypes in the digital form section.

Umami taste is an important target trait for bottle gourd breeding. We have determined that free glutamate content serves as a key factor that confers umami taste in the bottle gourd by analyzing correlation between free amino acid content and the umami taste scoring^22^. In the GourdBase phenome database, the umami taste trait represented by the free glutamate content, as well as the content of 18 more different types of amino acids, were deposited in numeric form. The umami taste phenotypes in numeric form of the GourdBase phenome database holds 135 accessions, which were assayed in 2014 and 2015. The free amino acids content was affected by the environment, which led to the differences in the umami taste phenotypes between 2014 and 2015^22^.

#### Markers/QTLs Search

**Figure 6:**
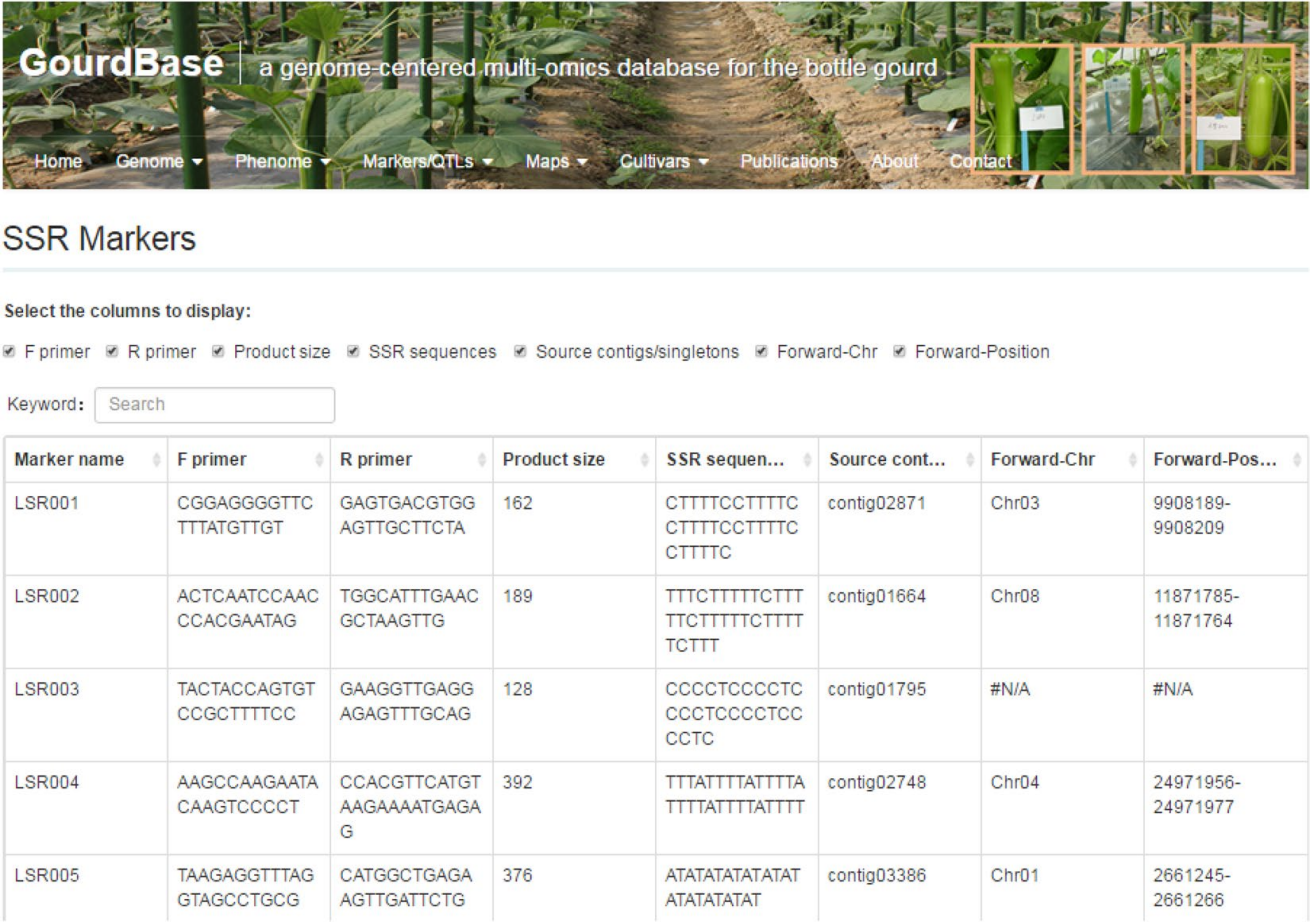
A snapshot of the SSR marker information window in the Markers/QTLs database.

The GourdBase markers/QTLs database collects over 6,000 molecular markers in various types including SNPs, Indels, SSR and AFLP markers, with key information such as source sequences, primer sequences, and product sizes that are readily retrievable (Fig. 6). This module allows users to search markers by type when dragging the mouse over the ‘Marker’ label. At present, all primers are displayed in tabular form. The markers/QTLs database also holds information on 17 published umami taste-related QTLs, 37 fruit shape-related QTLs and 2 bitterness taste-related QTLs. The QTL positions, left and right flanking markers, LOD value and PVE (%) are shown at present.

#### Genetic, physical and comparative genome maps

The GourdBase Map database contains two genetic maps, a genetic-physical correspondence map, and a genome comparative map between the bottle gourd and its relatives. One genetic map (the ‘JJ’ map) was constructed based on the 139-individual F_2_ population derived from the cross of the ‘Hangzhou gourd’ and ‘J129’ using SNP markers from RAD-Seq data^8^, and the other genetic map was constructed based on the F_2_ population derived from the ‘Hangzhou gourd’ and YD-4 cross using ddRAD-Seq data. The genetic-physical map correspondence information was previously published in Xu *et al*.^8^. Comparative genomic information (synteny maps) between the bottle gourd and its relatives (cucumber, watermelon and melon) have already been published^8^. Circos illustrations of the genome synteny are also available in this database.

#### Cultivars and publications

In addition to dedicated genomics-driven research on the bottle gourd, the ZAAS bottle gourd research program looks at the breeding of new cultivars for market use. During the past decade, a series of bottle gourd cultivars with elite fruit shapes and umami taste traits have been released, including ‘Zhepu’ No. 2, ‘Zhepu’ No. 6, ‘Zhepu’ No. 8 and ‘Zhepu’ No. 9. ‘Zhepu’ No. 2 bears long, straight fruit and is suitable for protected cultivation in the winter and early spring due to its strong growth potential, good fruit setting and early maturity. ‘Zhepu’ No. 6 bears beautiful, slender and straight fruit and is a highly resistant line to low temperatures and weak light. ‘Zhepu’ No. 8 bears medium-long straight fruit and is an elite, heat-resistant variety. ‘Zhepu’ No. 9, with its short straight fruit shape and strong umami taste, is suitable for cultivation during the summer and autumn. Detailed information and photos of the aforementioned bottle gourd cultivars are shown in the ‘Cultivars’ module of GourdBase.

The publications module of GourdBase currently harbors 18 publications supporting the aforementioned information can be freely read and downloaded with the authors’ permission. Among them, Xu *et al*. developed a Bayesian method for calling genotypes from an F_2_ population in the bottle gourd and constructed a high-density genetic map that provides a bright future of applying RAD-Seq to population genomic research for nonmodel species^8^. Xu *et al*. reported on the partial sequencing of the bottle gourd genome and revealed markers that are useful for phylogenetic analysis and breeding^25^. Wu *et al*. have determined free glutamate content to be a key factor conferring umami taste in the bottle gourd by genome-wide association analysis^22^.

## Discussion

The current version of GourdBase is v1.0. As more and novel omics data, such as re-sequencing, structural variation (SV), Chip-Seq and epigenomic data from the bottle gourd are continually available, GourdBase will always evolve over time. Future development will emphasize on the update, integration and centralization of the information to provide an easier and more comprehensive access to the large amount of data.

In the short term, more transcriptomics and expression data will be added continuously, and advanced functionality will be available gradually. For markers/QTLs database, a click-and-activation function will be incorporated to allow for the display of marker information along the genome in a graphic form. Currently, none of the maps allow one to click on a feature (*e*.*g*., a marker, gene, QTL) to obtain detailed information or external resources, and future development will add a greater number of feature types, more map sets, and a new search, display, and download options. In the long term, GourdBase is committed to building a complete and comprehensive information system that integrates spatial-temporal data of genomics, transcriptomics, proteomics, metabolomics, phenomics and more. With more cucurbits becoming available in the omics data, GourdBase will serve not only the bottle gourd research community but also researchers and breeders of the broader cucurbit crops.
